# To Examine the Horse’s Larynx

**Published:** 1890-02

**Authors:** 


TO EXAMINE THE HORSE'S LARYNX.—An invention likely to interest not only veterinarians but also those intrusted with the care of horses, has just been brought out at the Vienna Military Veterinary Institute. Prof. Polanski and Dr. Schindelka have constructed a laryngoscope admitting of the inspection by mirror of the horse's larynx. The apparatus is used with a tiny electric lamp, and the examination is made, not as heretofore, through the animal's mouth, but through its nostrils. The horses were easily controlled during the experiments which have been made at the Vienna Institute. The new method has the advantage of allowing the nostrils to be thoroughly examined at the same time as the larynx, which for certain diseases as glanders, collection of the sinuses, etc., is all-important.

